# Predictors of Patient Dissatisfaction with Services for Prevention of Mother-To-Child Transmission of HIV in Dar es Salaam, Tanzania

**DOI:** 10.1371/journal.pone.0165121

**Published:** 2016-10-21

**Authors:** Helga Naburi, Phares Mujinja, Charles Kilewo, Till Bärnighausen, Nicola Orsini, Karim Manji, Gunnel Biberfeld, David Sando, Pascal Geldsetzer, Guerino Chalamila, Anna Mia Ekström

**Affiliations:** 1 Department of Pediatrics and Child Health, Muhimbili University of Health and Allied Sciences (MUHAS), Dar es Salaam, Tanzania; 2 Department of Public Health Sciences, Global Health (IHCAR), Karolinska Institutet, Stockholm, Sweden; 3 School of Public Health and Social Sciences (SPHSS), Muhimbili University of Health and Allied Sciences (MUHAS), Dar es Salaam, Tanzania; 4 Departments of Obstetrics and Gynaecology, Muhimbili University of Health and Allied Sciences (MUHAS), Dar es Salaam, Tanzania; 5 Department of Global Health and Population, Harvard T.H. Chan School of Public Health, Boston, MA, United States of America; 6 Africa Centre for Population Health, Mtubatuba, South Africa; 7 Management and Development for Health (MDH) non-Governmental organization, Dar es Salaam, Tanzania; 8 Department of Infectious Diseases, Karolinska University Hospital, Huddinge, Stockholm, Sweden; International AIDS Vaccine Initiative, UNITED STATES

## Abstract

**Background:**

Mother-to-child transmission (MTCT) of HIV remains a major source of new HIV infections in children. Prevention of mother-to-child transmission of HIV (PMTCT) using lifelong antiretroviral treatment (ART) for all pregnant and breastfeeding women living with HIV (Option B+) is the major strategy for eliminating paediatric HIV. Ensuring that patients are satisfied with PMTCT services is important for optimizing uptake, adherence and retention in treatment.

**Methods:**

We conducted a facility based quantitative cross-sectional survey in Dar-es-Salaam, Tanzania, between March and April 2014, when the country was transitioning to the implementation of PMTCT Option B+. We interviewed 595 pregnant and breastfeeding women living with HIV, who received PMTCT care in 36 public health facilities. Predictors of overall dissatisfaction with PMTCT services were identified using a multiple logistic regression.

**Results:**

Overall 8% of the patients expressed dissatisfaction with PMTCT services. Patients who perceived health care workers (HCW) communication skills as poor, had a 5-fold (OR 4.9, 95% CI 1.8–13.4) increased risk of dissatisfaction and those who perceived HCW capacity to understand client concerns as poor, had a 6-fold (OR 5.7, 95% CI 2.3–14.0) increased risk. Having a total visit time longer than two hours was associated with a 2-fold increased risk of being dissatisfied (OR 2.3, 95% CI 1.1–4.7). Every 30-minute increment in total visit time was associated with a 10% higher (OR 1.1, 95% CI 1.0–1.2) risk of being dissatisfied. The probability of being dissatisfied ranged from 4% (95% CI 2% - 6%) in the presence of patient-perceived good communication, good understanding of patient concerns, and a total visit time below two hours, to 70% (95% CI 47% - 86%) if HCW failed in all of these aspects.

**Conclusion:**

Patient dissatisfaction with PMTCT services was generally low; reflecting that quality of care was maintained during Tanzania’s transition to Option B+ strategy aiming to increase the number of women initiating life-long ART in PMTCT clinics. Improved HCW communication with clients, their understanding of patient concerns and a reduction of the total visit time would further optimize women’s overall satisfaction with PMTCT services in Tanzania.

## Introduction

Mother-to-child transmission (MTCT) of human immunodeficiency virus (HIV), accounts for over 90% of new HIV infections among children [1]. Prevention of mother-to-child transmission (PMTCT) of HIV, using antiretroviral therapy (ART) throughout the breastfeeding period is a proven, efficacious intervention, which can lead to near elimination of MTCT (EMTCT) [2].

The ART uptake for PMTCT in sub-Saharan Africa is influenced by multiple factors operating at the individual, community or health facility levels [3, 4]. At the facility, in addition to the shortage of health care workers (HCW), poor patient-provider interaction contributes to dissatisfaction and eventually defaulting from PMTCT services [5, 6].

Patient satisfaction can be defined as the perceived fulfillment of patient needs and desires through the delivery of health care [7–9], or when patients perceive satisfactory quality of care and services that meets their expectations [10–12]. Despite problems with establishing a common definition of “patient satisfaction with care”, the concept continues to be widely used [9]. Many health systems frameworks, such as the WHO’s building blocks and the “control knobs” framework [9], include a measure of patients’ subjective evaluation of health services, such as “patient satisfaction” [13] or “responsiveness” [9] as an intrinsic health systems goal.

Hence, a health systems goal for scaling-up Option B+ efforts should focus not only on increasing the number of patients enrolled on life-long ART, but also on aspects of treatment delivery that influence patient satisfaction with care and long-term retention.

The introduction of ART for pregnant and breastfeeding women (Option B and B+) has contributed to a 3-fold reduction of MTCT rates (from 18% in 2010 to 6% in 2015) in eastern and southern Africa [14].

To achieve the 2020 fast-track targets to end AIDS among children, adolescents and young women, the number of new HIV infections among children needs to be reduced to below 40,000 infected infants by 2018 and fewer than 20,000 by 2020[15]. To achieve this, the implementation of Option B+ must reach all pregnant and breastfeeding women living with HIV and be sustained over time. However, there are indications that not all women diagnosed with HIV during pregnancy may be ready for lifelong ART and that a majority drops out of the program before the end of the breastfeeding period [16–18]. Studies have shown that, patient satisfaction with services can influence the retention in clinical care and adherence to lifelong ART, i.e. the major prerequisites for improved PMTCT outcomes [5, 19, 20]. For instance, patient satisfaction with HIV care has been linked to viral suppression [21]. Thus, optimizing patient satisfaction with PMTCT services appears crucial for retention in care throughout the PMTCT /EMTCT cascade and subsequently better outcomes [5, 22, 23].

Hence, patient satisfaction may be a feasible strategy to retain women and their families in care to achieve the benefits of PMTCT Option B+.

This study aimed to assess patients’ dissatisfaction with PMTCT services and related factors in public health facilities in Dar es Salaam Tanzania.

## Methods

### Design

A facility-based cross-sectional survey was conducted in Dar es Salaam, Tanzania between March and April 2014.

### Study setting

This study was conducted in public health facilities sampled from all public-sector providing PMTCT services in two of the three districts (Ilala and Kinondoni) in Dar es Salaam [24]. The two districts represents the smallest (Ilala) and the largest (Kinondoni) of the three districts of Dar-es-Salaam in terms of the population and number of health facilities [24]. At the time of the study, Tanzania was in the process of phasing out Option A (antenatal and intrapartum ART prophylaxis and nevirapine for HIV-exposed infants) and adopting Option B+ [25, 26]. PMTCT services in Tanzania are provided free of charge and follow the National guidelines that are regularly revised to match the WHO guidelines updates [27].

### Study population and sampling

All pregnant and breastfeeding women living with HIV, who were receiving PMTCT services in public health facilities within Ilala and Kinondoni districts in Dar-es-Salaam were eligible.

Out of 150 health facilities in these two districts (49 in Ilala and 101 in Kinondoni) providing the integrated antenatal care (ANC) and PMTCT services, we excluded 92 non-public facilities. Ten public facilities were further excluded due to lack of permission from authorities to access them. From the remaining public facilities, we selected all 18 facilities from Ilala and 18 matching (on facility level and size in terms of patient visits) facilities from Kinondoni. Given the length of the questionnaire, one interviewer could efficiently complete at least 3 interviews per day. At the facility, each day the interviewers listed the names of the patients seeking PMTCT services, then used simple random sampling to select 3 patients from this list and invited them to participate in an interview. If there were no patients registered for PMTCT services on the initial visit, the interviewer revisited the facility at least two more times.

### Data collection

Twenty research assistants with medical backgrounds were recruited and trained to use the research protocol and provided with the tools for data collection. The questions used to collect information on patient satisfaction were previously validated in other studies in Tanzania [5, 28, 29] and reflect the domains of health system responsiveness as defined by the WHO [30]. The questionnaire was translated from English to local language (Kiswahili) and back translated to English. Thereafter, the Kiswahili version of the questionnaire was piloted to ensure reliability, content validity, and quality of the questions before initiating data collection. Permission to conduct interviews in the health facilities was obtained from the district and health facility authorities. Written informed consent was obtained from all women prior to the interviews.

At the selected clinics, pregnant and breastfeeding women living with HIV were interviewed when exiting the clinics after receiving PMTCT services. Relevant information collected included: Patient and health facility characteristics, overall satisfaction with PMTCT services and patient-perceived quality of their interaction with health care workers. The interviewers filled in basic information on health facility characteristics. Respondents were asked to rate their overall satisfaction with PMTCT services on a Likert scale with 5 items ranging from “very good”(1) to “poor”(5). Then they were asked to rate the quality of their interaction with HCW on a Likert scale with 5 items ranging from “very good”(1) to “poor”(5), with regard to their perception on the HCW promptness of attention, clinical skills, communication skills, ability to listen to patients, understood patients concerns and maintaining the confidentiality of patient information. The women were also asked to rate, on a Likert scale with 3 items ranging from “most of the time”(1), “sometimes”(2), “no/never” (3), if they thought the HCW explained things to them clearly and encouraged questions.

To make sure all patients interpreted the questions similarly, the concepts of good communication, confidentiality and prompt attention were first defined in the questionnaire. Good communication was defined as when the provider listens carefully, explains things so that patients can understand, and allow enough time for patients to ask questions. Confidentiality of information was defined as when a patient’s medical information is kept confidential, and that patients can talk with the HCW without other people being able to hear the conversation. Prompt attention was defined as when patients had short waiting times for appointments and could get tests done quickly.

A compensation of 5,000 Tanzanian shillings (equivalent to 3.1 USD according to 2014 exchange rate) was given for respondent’s time after completing the survey. The respondents did not get information that they would receive compensation before giving consent. Strict confidentiality was observed, whereby the patient’s name and clinic registration number was not used on the questionnaire. Instead all questionnaires were assigned a special identification number. Completed questionnaires were stored in a locked metal filling cabinet and the electronic database was stored in password-protected file to which only the research team had access.

The Research and Ethics Committee of Muhimbili University of Health and Allied Sciences (MUHAS) Tanzania, approved the study on February 17^th^ 2014.

### Statistical analysis

For the purpose of this analysis, responses for our dependent variable (overall satisfaction with PMTCT services) were converted from a 5 items Likert scale to a binary outcome of “dissatisfied” (including “neutral”/ “fair”/ “poor) versus “satisfied” (including “very good” and “good”). The outcome of interest was being “dissatisfied” with PMTCT services.

Similarly the responses for our independent variables were converted from a 5 items Likert scale to a binary outcome, dividing the responses into “poor” (including “neutral”/ “fair”/ “poor), versus “good” (including “very good” and “good”). The independent variables were patient ratings of their perceptions with regard to HCW promptness of attention, clinical skills, communication skills, listening ability, understanding patient concerns and confidentiality with patient information. The other independent variables (patients rating of how often the HCW explained things clearly or entertained questions) were in a 3-item Likert scale and were categorized as “YES” (including “most of the time” and “sometimes”) and “NO”.

The associations between patients’ overall dissatisfaction with PMTCT services, and, health facility characteristics, patient socio-demographics, and patient-perceived quality of their interaction with HCW were tested using Fisher’s exact test.

Independent variables that were significantly associated with overall dissatisfaction with PMTCT services were used to create the final multiple logistic regression models using a backward elimination method.

We also examined possible multiplicative interactions between predictors in the final multiple logistic regression model by adding their product terms. A formal p-value for departure from interaction was obtained using a likelihood ratio test comparing the maximized log-likelihoods of the model with and without the product terms.

To examine the variation of the probability of overall dissatisfaction with PMTCT services in relation to possible predictors, we used multiple logistic regression models and reported the odds ratios (ORs) and 95% confidence intervals. “Total visit time” was modeled as a continuous quantitative predictor assuming linearity. We also examined potential departure from linearity using restricted cubic splines with 3 knots at fixed percentile of its distribution [31]. Statistical analyses were performed using Stata software version 14 (Stata Corp 2014 Stata Statistical Software).

## Results

**Fig 1:**
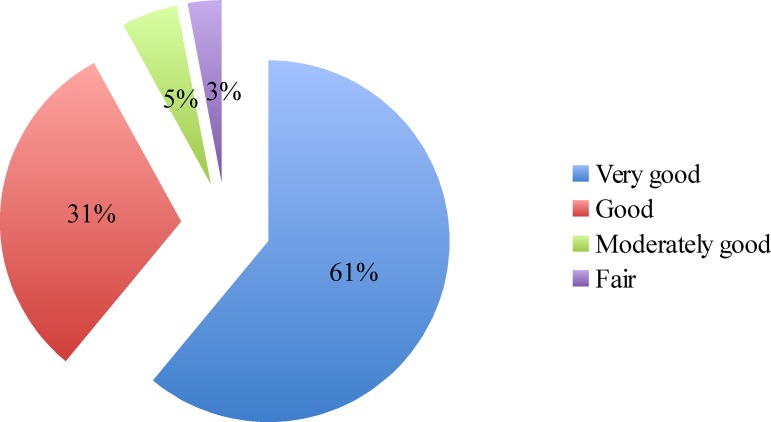
Distribution of the overall patient satisfaction with PMTCT services. Color fill represents levels of dissatisfaction as rated by patients; blue represents “very good", brown represents “good, green represents “moderately good" and purple represents “fair" satisfaction.

Six hundred women who were receiving integrated ANC/PMTCT services from 36 different public health facilities in two out of three districts of Dar es Salaam were eligible and 595 (99%) gave consent to be interviewed. Overall, only 48 (8%) of the women reported dissatisfaction with PMTCT services (3% and 5% who rated services as fair and poor, respectively). However, the majority, 360 (61%) rated services as good and 187 (31%) rated PMTCT services as very good (Fig 1).

The mean age of the respondents was 30 (±5.3) years and the average number of children per woman was 2 (±2) children. The majority, 70%, had a primary school education, 20% had secondary school education or more, while 10% had never been to school. Slightly less than two-thirds (63%) of these pregnant and breastfeeding women were in a formal relationship (married or cohabitating) while the rest were divorced, single or widowed. None of these factors were significantly associated with the overall dissatisfaction with the PMTCT services (Table 1).

**Table 1:** Patient characteristics according to their dissatisfaction with PMTCT services (N = 595).

	Total N	Dissatisfied %	p-value
**District**			
Ilala	199	11%	0.78
Kinondoni	396	7%	
**Age (years)**			
<25	108	8%	
25–30	196	7%	0.66
>30	291	9%	
**Parity**			
≤2	332	7%	
>2	192	8%	0.81
**Education**			
Not been to school	60	8%	
Primary Education	421	8%	1.00
Secondary Education and above	114	8%	
**Occupation**			
Not employed	123	6%	
Formal employment	65	12%	0.43
Self-employment	187	8%	
Housewife	211	7%	
**Marital status**			
Single	58	5%	
Married/cohabiting	367	9%	
Divorced	145	4%	0.26
Widowed	11	18%	
**Travel time to clinic**			
<15 minutes	99	9%	
15–30 minutes	204	5%	
31–60 minutes	165	9%	0.53
>60 minutes	121	9%	
**Disclosed HIV status**^**(**^^**a**^^**)**^			
Yes	488	7%	
No	74	11%	0.22
**Year of HIV diagnosis**			
≤ 2012	252	8%	
2013	200	7%	0.82
2014	105	7%	

Total counts do not always add up to *N* = 595 because some variables have missing data

^a^ Disclosed HIV status to any family member

The household expenditures for the majority of the women interviewed were below or just above the poverty line of approximately 2 USD/person/day. The majority were housewives or lacked a formal job, but 43% were either employed or self-employed. Only 19% of women had been diagnosed with HIV and received PMTCT services for less than one year before the interview. Furthermore, the majority (86%) of women had disclosed their HIV status to at least one family member before the interview. None of these factors was significantly associated (all p>0.05) with patients’ overall dissatisfaction with the PMTCT services (Table 1).

Nurses attended three quarters of all the patients and 70% of women received services in lower level facilities (dispensaries). The majority (91%) of the women reported that facility-opening hours were convenient and 71% said that PMTCT services met their expectations. For 74% of the women, the time between entering and exiting the facility was less than 2 hours. Among all the above-mentioned factors, only a total visit time of more than 2 hours (p >0.001) was significantly associated with patients’ overall dissatisfaction with PMTCT services (Table 2).

**Table 2:** Patient ratings of their dissatisfaction with PMTCT services according to health facility characteristics.

	Total N	Dissatisfied %	p-value
**Level of health facility attended**			
Hospital/ health center	181	6%	0.24
Dispensary	414	9%	
**Main care provider**			
Doctors	127	7%	
Nurses	449	8%	0.70
Others (counselors or pharmacists)	9	11%	
**Services met client expectations**			
Yes	427	8%	0.15
No	16	19%	
**Opening hours convenient**			
Yes	543	7%	0.51
No	38	11%	
**Total visit time (hours)**			
<2	384	5%	
≥2	141	13%	0.01
**Patient perception of HCW**			
**Clinical skills**			
Good	557	6%	
Poor	32	31%	<0.001
**Promptness of attention**			
Good	552	6%	
Poor	37	32%	0.001
**Confidentiality level**			
Good	550	6%	
Poor	37	27%	0.001
**Understanding patient concerns**			
Well	541	5%	
Poor	47	40%	<0.001
**Often entertaining questions**			
Yes	397	5%	
No	189	13%	0.002
**Often explaining things clearly**			
Yes	440	6%	0.001
No	148	14%	
**HCW listened to patient**			
Well	568	7%	
Poor	20	35%	<0.001
**Communication with patient**			
Good	554	6%	<0.001
Poor	32	41%	

Total counts do not always add up to *N* = 595 because some variables have missing data

Most of the women reported that they had a good perception of the quality of HCW’s communication with the patients (94%), the promptness of attention (94%), the confidentiality level and clinical skills of the HCW (94%). Similarly, 92% and 96% of the patients, respectively, felt that HCW did understand their concerns and listened well to patients. Fewer patients thought that HCW often entertained questions (68%) and 74% found that HCW often explained things clearly. The poor rating on patient-perceived HCW communication skills, promptness of attention, confidentiality with patient information, clinical skills, understanding patient concerns and listening well to patients were significantly associated (all p<0.05) with overall patient dissatisfaction with PMTCT services. Similarly, patient who thought that HCW did not often entertain questions or give clear explanation, were significantly (p<0.05) more likely to report overall dissatisfaction with PMTCT services (Table 2).

The average visit time was 1.8 (SD = 1.7) hours, and the difference between the satisfied and the dissatisfied categories was 0.82 hours (95% CI 0.24–1.39, p = 0.005).

In univariable analysis, the unadjusted odds of dissatisfaction were high and significant for patient-perceived poor HCW communication skills, promptness of attention, confidentiality with patient information, clinical skills, understanding patient concerns, listening to patients and not often willing to entertain questions or explain things clearly to patients (Table 3).

**Table 3:** Odds ratios and 95% confidence intervals of dissatisfaction with the PMTCT services according to the patient’s perception of care received.

Variables	Univariable	^1^Multivariable	^2^Multivariable
Patient perception of HCW’s	OR (95% CI)	OR (95% CI)	OR (95% CI)
Poor understanding of concerns	13.4 (6.6–27.2)	4.3(1.4–13.1)	5.7(2.3–14.0)
Poor communication with patients	11.1 (5.1–24.6)	3.6 (1.2–10.8)	4.9 (1.8–13.4)
Patient total visit time ≥ 2 hours	2.5 (1.3–4.9)	2.3 (1.1–4.9)	2.3 (1.1–4.7)
Poor promptness of attention	7.5 (3.5–16.4)	1.2 (0.3–4.6)	-
Poor confidentiality	5.6 (2.51–12.6)	1.1 (0.3–3.6)	-
Poor clinical skills	6.8 (3.0–15.4)	1.6 (0.5–5.5)	-
Not willing to entertain questions	2.6 (1.4–4.8)	1.0 (0.4–2.6)	-
Not explaining things clearly	2.9 (1.5–5.3)	1.2 (0.5–3.2)	-
Poor listening to patient concerns	7.5 (2.8–19.9)	1.6 (0.4–7.2)	-

^1^ Multiple logistic regression models including all the potential predictors of dissatisfaction.

^2^ Multiple logistic regression models including the three strongest predictors of dissatisfaction.

A multivariable analysis, including simultaneously all the patient ratings, suggested three strong and significant predictors of being overall dissatisfied with PMTCT services: total visit time exceeding 2 hours, patient-perceived poor HCW communication skills and understanding of patient concerns.

Time exceeding 2 hours was associated with 2-fold higher odds of dissatisfaction (OR 2.3, 95% CI 1.1–4.7). Patient-perceived poor HCW communication skills resulted in 5-fold higher odds of dissatisfaction (OR 4.9, 95% CI 1.8–13.4). Poor understanding of patient concerns was associated with 6-fold increased odds of dissatisfaction (OR 5.7, 95% CI 2.3–14.0) (Table 3). Further adjustment for age, marital status, educational level, and health care worker cadre and facility level did not substantially change the estimates.

**Fig 2:**
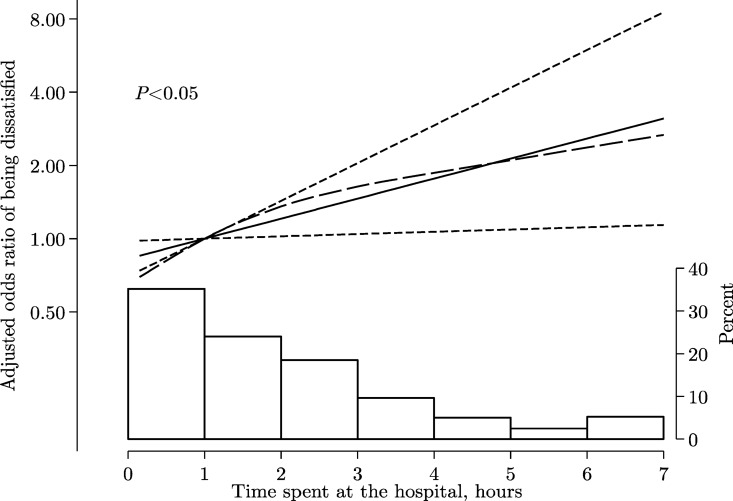
Odds ratio of being dissatisfied with the PMTCT services according to time spent at the hospital (hours). Data were fitted using a multiple logistic regression model adjusting for patients perceived poor health care workers’ communication skills and understanding of patients concerns. Solid line is a linear trend, dashed lines are 95% confidence limits of the linear trend, and the long-dash line is the curvilinear trend estimated with restricted cubic splines.

In our final model, we modeled total visit time as a continuous predictor and found no evidence of departure from linearity (p-value for non-linearity = 0.52). Every 30 minutes’ increment in total visit time was associated with 10% higher odds (95% CI 1.01–1.20) of being dissatisfied (Fig 2).

The probability of being dissatisfied with PMTCT services varied considerably according to patient-perceived HCW communication skills, understanding patient concerns, and total visit time. It ranged from 4% (95% CI 2% - 6%) in the presence of patient-perceived good communication, good understanding of patient concerns, and, a total visit time of less than 2 hours, up to a 70% (95% CI 47% - 86%) probability of being dissatisfied with PMTCT services when all these three factors failed to be fulfilled.

We also assessed possible departure from interaction and we found no evidence of interaction between patient-perceived poor communication skills and poor understanding of patient concerns (p = 0.67), patient-perceived poor communication skills and total visit time (p = 0.34) or poor understanding of patient concerns and total visit time (p = 0.50).

## Discussion

Overall, only a small proportion of pregnant and breastfeeding women living with HIV in Dar es Salaam, Tanzania reported dissatisfaction with PMTCT services. However, low rates of patient dissatisfaction overall do not necessarily mean that the quality of PMTCT services is not sometimes compromised [32]. For instance, despite the low level of dissatisfaction, we identified three strong and significant predictors of overall dissatisfaction with PMTCT services; patient-perceived poor HCW understanding of concerns, patient-perceived poor HCW communication skills and a total clinic visit time exceeding two hours being associated with a 5-fold, 4-fold and 2-fold increased risk of being dissatisfied with PMTCT services, respectively. Whereas combined, the individual effects of these factors magnified the probability of being dissatisfied with PMTCT services up to 70%.

Our results also show that these identified predictors influenced women’s dissatisfaction with PMTCT services equally regardless of individual or health facility characteristics. This implies that workforce development to ensure high quality patient-provider interactions where service providers can communicate well with their patients, as well as are able to show a good understanding of their patients’ concerns is very important to keep women living with HIV satisfied with PMTCT services. Strengthening health systems to reduce patients’ visit time will be another crucial strategy for effective scale-up of high-quality PMTCT services.

Despite existing time constraints and staff shortages [33], that compromise HCWs’ ability to provide good care [34], our results indicate that PMTCT providers, succeed to provide services that meet expectations of the majority of the patients to a very large extent. We cannot entirely exclude the possibility that the high ratings may reflect a fear among women to rate the services as poor, especially since most of them rely on the same facilities for their subsequent care. However, we made a thorough attempt to reduce the risk of reporting bias through the use of independent observers and interviewers, and, anonymous questionnaires. Furthermore, our findings are plausible, and consistent with regard to the levels of satisfaction with PMTCT and HIV services reported in other low-income countries [5, 29, 35–37].

Patient satisfaction with PMTCT and ART services, is important as it influences the uptake, retention and adherence [4, 20, 38], hence, patient satisfaction often serves as a determinant of adequate viral suppression [21]. Thus, although only a few patients were dissatisfied with the PMTCT services they received, long-term retention and adherence to lifelong ART (Option B+), will likely require additional efforts [5, 35, 39–41], in particular with regards to ensuring high-quality patient-provider interactions[3, 4]. Our findings also raise concerns that the information women receive with regards to the aim and expected procedure of PMTCT could be less than optimal, because a fairly large proportion of HCW did not often encourage questions from the patients or explained things clearly. This is likely to impact both adherence and retention in ART, the key elements of the overall effectiveness of PMTCT interventions especially Option B+, where patients clearly need to understand the information provided regarding their HIV diagnosis to be able to accept and discuss their status with their partner or other close family members before they are initiated on life-long ART[17]. A study in Ethiopia reported that only one third of women received and understood the messages related to MTCT and PMTCT [35]. Previous research has also found that poor patient-provider interaction in terms of unclear communication, confidentiality concerns, and patients not feeling comfortable to ask questions are reasons for missing PMTCT services [6] and that sub-optimal patient-provider interactions can be significant barriers to accessing ART for pregnant and post-natal women [42].

We previously found that as much as 8 out of 10 new mothers in Dar-es-Salaam who needed ART for their own health (CD4 count <200 cells/mL) were viremic after the breastfeeding period, raising very serious concerns about the feasibility of life-long adherence to ART in this population [43]. We speculate that part of this very poor adherence to ART post-natally, could be due to unclear communication in early patient-provider interactions. Our speculation are based on the fact that the new mothers reported that they had lost their motivation to continue taking ART after knowing they had protected their children from HIV, because of fearing side effects, or, because they did not feel ready for life-long medication [16]. Good provider-client communication could reduce the risk of defaulting and has previously been found to have a positive effect on patient satisfaction with PMTCT services [6, 35, 44, 45]. Clear communication positively influences the uptake of PMTCT and ART services including adherence to visits and medicines [6, 28]. A previous study involving private HIV clinics in Dar es Salaam found an independent effect of confidentiality and promptness of attention, on patient satisfaction with ART services [29]. This is probably because patients more easily can be noticed and stigmatized in clinics where only people living with HIV attend, compared to integrated ANC/PMTCT clinics where all pregnant and breastfeeding women go, regardless of their HIV status. Additionally, patients attending private facilities may also have higher expectations that such settings should be able to provide timely services, optimal privacy and confidentiality given the lower work load in private compared to public facilities [4]. However, confidentiality and privacy is important for patients’ satisfaction with ART and PMTCT services, both involving counseling and testing for HIV, and many patients are worried about accidental disclosure of their HIV status [5, 6, 35].

Findings from our study also indicate that overall dissatisfaction with PMTCT services increased with a low rating of perceived clinical skills among the HCW. Although this effect was not statistically significant, it can affect the uptake of antiretroviral prophylaxis among pregnant and breastfeeding women living with HIV who attend ANC clinics [4].

Finally, long waiting/visit time has also previously been shown to reduce patient satisfaction in PMTCT services and subsequent utilization of ANC/PMTCT services [4, 5, 42, 46–48]. Long visit time at the clinic is usually a result of high patient load in relation to the scarce human resources available to meet this demand, in turn influencing the quality of care [46]. Here, our results have several interesting implications since neither the level of health facility, nor cadre, influenced patient dissatisfaction with PMTCT. This opens up for task-shifting as an opportunity to reduce staff workload and subsequently ensure quality patient-provider interactions.

This study had some limitations. Firstly the cross-sectional study design does not allow for a causal effect analysis, but it is nonetheless useful for identifying predictors of patient dissatisfaction with PMTCT services in Tanzania. Secondly, despite the precautions taken in terms of external interviewers and anonymity, some respondents may not have been entirely open regarding the perceived quality of their interaction with HCW, and that could result in information bias. Thirdly, it is difficult for patients to accurately measure actual clinical skills or communication skills of the HCW. However this study aimed to gather the patients’ own views and perceptions of services as subjective indicators of quality of care. Finally, our findings may also have limited generalizability to private facilities and other PMTCT facilities in Tanzania since we only used public facilities in Dar es Salaam.

Despite these limitations, we believe this study gives a good picture of the level of dissatisfaction with PMTCT services as well as possible predictors in this East-African setting. It is consistent with other studies in low-income countries and reflects that the struggle of HCWs to offer services and meet expectation of patients in PMTCT facilities is successful in spite of a high workload and often poor remuneration.

## Conclusion

Patient dissatisfaction with PMTCT services was generally low. However, improving the quality of communication with patients, understanding patients’ concerns and reducing the total visit time is probably a strategic move to improve client satisfaction with PMTCT care and in turn optimizing the likelihood of retention in PMTCT Option B+ programs. This is crucial in our strive towards EMTCT, and to achieve the fast-track targets to end AIDS among children, adolescents and young women.
